# The promise and peril of mirror bacteria

**DOI:** 10.3389/abp.2026.16673

**Published:** 2026-05-29

**Authors:** Kyna Rigouts, Gregor Becker

**Affiliations:** 1 Faculty of Biochemistry, Biophysics and Biotechnology, Jagiellonian University, Kraków, Poland; 2 Doctoral School of Exact and Natural Sciences, Jagiellonian University, Kraków, Poland

**Keywords:** bioethics, experimental ethics, mirror bacteria, mirror life, synthetic biology

## Abstract

Mirror bacteria are organisms constructed from mirror-image biomolecules unlike those used by all known life and are moving from theoretical concept to practical feasibility. Their unusual chemistry that makes them resistant to natural degradation and isolated from ecosystems, fuels optimism for applications such as more stable medicines, long-term information storage, and novel platforms for bioengineering. At the same time, these very characteristics raise reservations: they challenge existing definitions of “life,” expose gaps in regulation, and risk undermining public trust if deployed without careful oversight. This perspective assesses not only those risks but also the opportunities of mirror bacteria, moving beyond narrow biosafety debates to explore their broader ethical, regulatory, and cultural implications. We argue that mirror bacteria represent a rare testcase for how science and society navigate the responsibilities of creating fundamentally new forms of life. Their development offers not only technical possibilities but also a chance to build more resilient frameworks for governance and ethics in synthetic biology.

## Introduction

**FIGURE 1:**
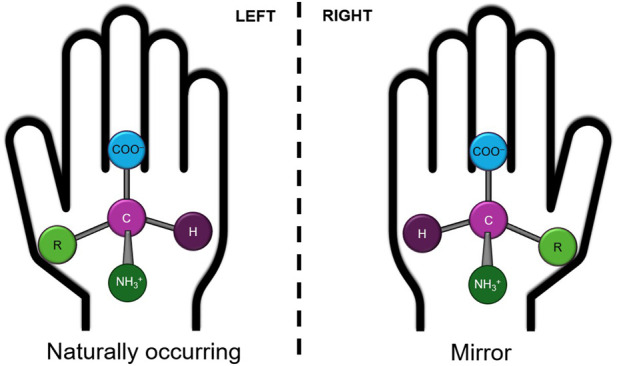
Fundamental model for chiral comparison of L-amino acid (naturally occurring) and D-amino acid (mirror amino acid).

Mirror bacteria (MB), a microbial-scale subset of the broader concept of mirror life (system-level chirality inversion), represent a groundbreaking frontier in synthetic biology, characterized by their unique biochemical makeup (Adamala K. et al., 2024; Adamala K. P. et al., 2024). Unlike conventional organisms, which rely on molecules of a specific chirality such as L-amino acids and D-nucleotides, MB are made up from the mirror images (Figure 1). Although fully functional MB have not yet been realised, early experimental approaches already point toward their development, including the synthesis of mirror peptides and nucleic acids, cell-free protein synthesis systems (such as the modified protein synthesis using recombinant elements (PURE) system), and emerging work on mirror enzymes (Peplow, 2016; Wang et al., 2016; Qi et al., 2024; Cui et al., 2022).

As MB move closer to practical realization, they bring with them both exciting opportunities and important risks. So far, much of the discussion – most notably in the recent paper *Confronting risks of mirror life* and its accompanying technical report – has focused on the potential dangers, especially biosafety concerns (Adamala K. et al., 2024; Adamala K. P. et al., 2024).

Even though MB are biochemically isolated from natural ecosystems, their behaviour in laboratory or clinical environments is still uncertain, raising difficult questions about containment and safety (Adamala K. et al., 2024; Adamala K. P. et al., 2024). At the same time, their unusual properties inspire optimism in certain areas. The resilience of mirror molecules to natural enzymatic degradation could open new possibilities in medicine, such as more stable drugs or advanced diagnostic tools.

These developments also spark debate over regulation. Regulatory debates have centred on whether current legal and ethical frameworks are sufficient to govern organisms that exist entirely outside natural evolutionary processes.

This paper looks beyond these initial concerns to explore not only the underexamined threats, but also the opportunities MB present. By examining both the promise and peril embedded in MB, we aim to promote a deeper, more nuanced dialogue about the future of life sciences in an age where biology is increasingly designed and engineered.

## Ethical risks

While past discussions have tackled aspects such as biosafety, biomedical applications and regulatory frameworks, they tend to focus on the immediate, practical issues related to the use of MB (Adamala K. et al., 2024; Cengel, 2023; Sample, 2024; Bureaubiosecurity, 2025; Epstein et al., 2025). In the following sections, we will delve into other underexamined dimensions.

### The concept of “life” and the possibility of escalation

Traditionally, “life” has been defined by certain characteristics such as metabolism, replication and responsiveness to stimuli (Epstein et al., 2025). All these traits are based on natural life. While MB could meet many of these criteria, their unique makeup puts them outside the natural evolutionary lineage of life. Because of that, placing MB in the category of “life” would blur the lines between the synthetic- and the natural-, the designed- and the evolved-, and the chemically-isolated and integrated organisms. Uncertainty where to place MB could disrupt environmental law and biomedical ethics as clear definitions are necessary in these kinds of fields. Legal frameworks overseeing biodiversity and biohazard regulation, for example, rely on shared understandings of what describes a living system.

The fact that MB are not clearly defined in fields like ethics and law could create regulatory loopholes and open the door for corporate exploitation. For example, a company could argue that MB are not “alive” by traditional standards and therefor do not fall under existing laws. The lack of regulation could lead to the release or commercialisation of MB with minimal oversight.

This kind of intensification is not without any precedent. Both nuclear energy and gene editing started out as highly specialized, seemingly controllable innovations that were confined to laboratories or narrow applications. Yet over time, they created widespread societal and ethical challenges that went well beyond their original contexts. What connects these two innovations is the shared trajectory. Early optimism and restraint eventually gave way to broader use, bringing both unforeseen challenges and transformative benefits. For example, gene editing has revolutionized medicine, and nuclear power continues to play a role in low-carbon energy strategies.

This dual legacy shows that ethical foresight is not about resisting innovation, but about preparing for its complex, real-world consequences. Because, as complexity increases, our ability to predict outcomes often decrease. And if ethical reflection fails to keep pace with innovation, society could find itself crossing borders without ever having stopped to think where they should have been in the first place.

These doubts and the potential for corporate misuse risk eroding public trust in science and policymaking, making it more difficult to build consensus around the responsible use of emerging biotechnologies. At the same time, if MB are classified as “alive,” the definition of life could become so broad that it loses much of its conceptual force, weakening arguments that depend on that very concept.

While the production of MB is a clear display that humanity can create entirely new systems, the threat of a slow drift in ethical standards increases once MB are actually made. That is because as each step in the creation of MB becomes normalized, the line for what counts as radical or risky moves further away. If the creation and use of MB become widely accepted as safe and ethically uncontroversial, then this could reduce resistance to more complex interventions such as designing entire artificial ecosystems. That lack of resistance could pave the way for more radical biotechnological interventions to become normalized without the necessary scrutiny they deserve.

### Intellectual overreach and translational misjudgement

Even if reflection and regulation keep pace with the creation of MB, MB’s ability to mimic certain biological behaviours could still lead to their results being mistakenly treated as equivalent to those from natural life. However, behaviour does not always equal function, nor is it based on the same chiral building blocks as natural life. This kind of translational fragility could be especially problematic in fields like drug development, where decisions based on flawed prognoses could carry real-world consequences.

### Erosion of ecological uniqueness

The existence of MB could impact how we symbolically and aesthetically value the planet’s natural systems, even though they do not directly interact with Earth’s ecosystems. That is because Earth is not seen merely as a collection of living things, but a unique, unified system that is shaped by its specific chemistry and evolutionary history. That particular chirality of Earth’s life adds to its moral and aesthetic significance, much like how untouched wilderness or endangered cultures are valued. While concepts like morality and beauty are not inherent in biology itself, they are important human ways of interpreting and appreciating natural systems.

MB could challenge the idea of Earth’s evolutionary uniqueness and wholeness. Even if kept isolated, the presence of MB adds complexity to the definition of the biosphere, potentially fragmenting it in a way similar to how synthetic buildings or structures can be seen as disrupting the natural beauty of remote landscapes (Guo et al., 2021; Summerson and Bishop, 2012). Moreover, the ability to engineer alternative life forms might weaken the argument that Earth’s biochemical heritage is irreplaceable, which could undermine conservation efforts that rely on the belief that natural systems are unique and must be preserved.

## Opportunities

Even though caution is necessary for MB, their very features also open doors for new opportunities. By existing on the fringes of our current understanding of life, MB challenge anthropocentric assumptions, encourage a deeper investigation of the ethics of “artificial biological” design, and even create new spaces to rethink care, stewardship, and humility in science. Instead of purely pushing against moral and conceptual limits, mirror life could offer an opportunity to reshape those boundaries, improving rather than diminishing ethical discourse.

### Experimental ethics

Previously, it was thought that MB could offer a distinctive, though contested, opportunity for experimental biology because of their reversed biochemical makeup and resulting incompatibility with natural life (Schmidt, 2010). In theory, their inability to metabolize natural organic material or exchange genes could create a kind of “ethical buffer zone,” enabling research on high-risk ideas without directly endangering natural ecosystems. This idea is consistent with established biosafety approaches such as metabolic compartmentalisation and genetic firewalls (Bar-Peled and Kory, 2022; Schmidt and Kubyshkin, 2021). For example, designing organisms with dependencies on non-natural, chirality-specific nutrients could quarantine MB’s survival to controlled environments. In parallel, the use of orthogonal genetic systems could reduce the risk of horizontal gene transfer by preventing functional compatibility with natural DNA and cellular machinery. This vision, which could be referred to as “contained ethics”, imagines MB as safe testing grounds for engineered symbiosis, synthetic gene drives, or stress tests for bioengineering resilience and planetary terraforming.

But in contrast to those theories, recent analyses, including the “*Technical Report on Mirror Bacteria: Feasibility and Risks*”, urge caution before treating biochemical isolation as a guarantee of safety (Adamala K. et al., 2024; Adamala K. P. et al., 2024). While MB cannot integrate into natural evolutionary pathways, their resistance to natural degradation could also make them unexpectedly persistent or difficult to control if containment fails. Moreover, even abovementioned safeguards could degrade over time through mutations, system leakage, or even incomplete orthogonality, which challenges assumptions of long-term containment. From this perspective, MB research requires the same, if not stricter, safeguards as work on natural organisms.

This apprehension creates a valuable ethical opportunity. By confronting the gap between theoretical containment and practical uncertainty, MB can serve as a test case for developing more robust ethical frameworks that actively interrogate and stress-test containment strategies before they are applied more broadly. In this way, MB could help build a more mature, precaution-informed approach to experimental ethics in synthetic biology, one capable of guiding research at the edges of life as we know it.

### Temporal life stewardship and biological memory

In addition to being an encouragement for developing frameworks, MB could become biological archives. That is because MB are more stable than regular organisms due to their high resistance to degradation by natural enzymes and environmental processes.

Their unique stability means MB could, in principle, act as long-term carriers of information, preserving for example historical records, knowledge, or genetic instructions across geological or even interstellar timescales that potentially survive events that would erase digital archives or natural life forms. For example, MB could store important information to be passed on to future generations, or even alien civilizations.

Using MB as long-term information storage raises important ethical questions: what information should we choose to preserve? Who gets to decide what is worth saving? And what responsibilities do we have when creating life designed to outlive us by centuries or millennia? These questions encourage us to rethink life as a medium for communicating across vast stretches of time and/or space, and to consider the legacy and meaning of such stewardship. Consequently, MB move synthetic biology’s role from short-term use to long-term guardianship, even if the practical realisation of such applications remains dependent on future technological progress.

### Expanding education and anthropocentric views

Because all known organisms on Earth use molecules with a specific chirality, biology has long assumed a single, universal biochemical foundation for life. MB show that life as we know it, is based on our current understanding and is just one of countless possibilities. This realization pushes us to rethink why life on Earth evolved as it did and to consider alternative (biochemical) possibilities and broaden how we think about life. MB can serve as educational tools that encourage more creative and flexible approaches to biology, synthetic biology and evolution by forcing students and researchers to move beyond traditional frameworks. The existence of MB also promotes interdisciplinary dialogue between different disciplines such as philosophy, chemistry, astrobiology, and ethics to explore bigger questions about life’s nature.

By expanding our concept of life beyond Earth-centred definitions, MB promote a less human-focused perspective in bioethics, which often value life based on its similarity to human biology, cognition, or experience, and invite us to reflect more deeply on our place within the living world (Zylinska, 2009). This broader, more inclusive perspective can then better prepare us to engage with potential future discoveries, whether alien life forms or radically engineered synthetic organisms. By expanding the ethical frameworks we use to understand and respect diverse forms of life, MB function not only as scientific novelties but also as powerful tools that challenge assumptions and enrich our understanding of what life can be.

## Discussion

MB stand at a threshold between the possible and the unprecedented, revealing the expanding scope of biology that moves beyond what nature has produced. Their reversed biochemical makeup challenges established definitions of life, tests the adequacy of existing frameworks, and provokes new ethical questions. They carry genuine risks that range from potential gaps in regulation, misunderstandings in application, changes in public trust, and questions about the value we place on natural systems – all of which demand require careful and ongoing attention. Yet these same characteristics open pathways to opportunities to expand ethical inquiry, explore new forms of biological stewardship, and deepen our understanding of life beyond traditional frameworks.

Because MB carry both promise and peril, the challenge is not to choose between optimism and caution, but to sustain both in equal measure. Treating MB as neither inherently dangerous nor inherently liberating allows us to approach them with the careful consideration they warrant. Their creation should not be seen as the endpoint of an engineering feat, but as the beginning of a long-term responsibility to integrate new life forms into all our current and future frameworks.

If synthetic biology is the art of rewriting life’s possibilities, then MB represent a chapter that must be written with foresight and care, fully aware that every line added changes the shape of the story. The measure of our success will not be how far we push the boundaries of what can be made, but how well we preserve the wisdom to decide when and why those boundaries should move at all.

## Data Availability

The original contributions presented in the study are included in the article/supplementary material, further inquiries can be directed to the corresponding author.
